# Rehabilitation and Exercise in Dermatomyositis: A Case Report and Narrative Review of the Literature

**DOI:** 10.7759/cureus.33034

**Published:** 2022-12-28

**Authors:** Duarte Calado, Frederico Moeda, Madjer Hatia, Sérgio Pinho, Marta Amaral-Silva

**Affiliations:** 1 Physical Medicine and Rehabilitation, Centro Hospitalar Lisboa Ocidental, Lisboa, PRT; 2 Physical Medicine and Rehabilitation, Centro Hospitalar Universitário de Lisboa Central, Lisboa, PRT

**Keywords:** nxp-2, idiopathic inflammatory myopathy, dermatomyositis, exercise, rehabilitation

## Abstract

Dermatomyositis (DM) is an idiopathic inflammatory myopathy most commonly characterized by proximal, progressive, symmetrical muscle weakness, as well as specific dermatological manifestations. The presence of nuclear matrix protein 2 (NXP-2) autoantibodies is predominantly associated with joint contractures and calcinosis. A 19-year-old female was diagnosed with DM with positive anti-NXP-2 autoantibodies. She had severe joint involvement of the shoulders, elbows, wrists, and ankles, and the presence of calcinosis was documented on radiographs. Concomitantly, she presented with heliotrope erythema on the eyelids and Grotton’s papules on the interphalangeal joints of the hands. After performing a diagnostic investigation and beginning targeted therapy, the patient was transferred to an inpatient Physical Medicine and Rehabilitation Department to carry out a rehabilitation program. The patient had a favorable outcome, with improved range of motion and muscle strength, with a Manual Muscle Testing 8 at the time of admission of 73/150, and at discharge from the hospital of 94/150. Regarding the functional scales, she had a Functional Independence Measure at the time of admission of 87/126 and a Barthel Index of 50/100, with an objective improvement at the time of discharge to 118/126 and 90/100, respectively.

DM is an insidious chronic disease with multisystemic involvement and can lead to a great loss of independence. Most patients with DM do not recover their previous muscle function, which leads to a negative impact on their quality of life. The institution of an early rehabilitation program seems to have beneficial effects on the functionality and independence of these patients. Its treatment is based on a multidisciplinary approach, and the established rehabilitation program must be individualized and directed to the deficits and limitations of each patient.

## Introduction

Idiopathic inflammatory myopathies are a heterogeneous group of rare multisystemic immune-mediated diseases with infiltration of skeletal muscle tissue by autoantibodies [1], with consequent fatigue and decreased muscle strength. This group includes dermatomyositis (DM), polymyositis (PM), inclusion body myositis (IBM), overlapping myositis, and autoimmune necrotizing myositis [2], which are often associated with other immune-mediated diseases and neoplasms.

DM affects predominantly females (ratio 2:1) [3], with a bimodal age distribution (5-15 years old and 45-65 years old) [4]. Autoantibodies are present in about 50% of cases of myositis, and the presence of anti-nuclear matrix protein 2 (NXP-2) autoantibodies is predominantly associated with joint contractures and calcinosis. The clinical course is characterized by an insidious onset of muscle fatigue, atrophy, and symmetrical muscle weakness (proximal muscles of the upper and lower limbs, cervical flexors, pharyngeal and respiratory muscles) [2], with specific dermatological manifestations [5] (Gottron’s sign and papules, heliotrope erythema, poikiloderma with “shawl” sign), as well as symmetric joint involvement (usually small joints). In more severe cases, it may also be accompanied by dysphagia with an increased risk of aspiration pneumonia, cardiovascular dysfunction (arrhythmias, myocarditis, endocarditis in the chronic setting) [1], and respiratory dysfunction (restrictive syndrome).

When possible, a skin biopsy should be performed to confirm a diagnosis of DM [5]. Serum increase in creatine phosphokinase (CPK) levels and pathological changes characteristic of myopathy in electromyography with nerve conduction studies may also be present [4]. Pharmacological treatment includes high-dose corticosteroid therapy (with progressive weaning depending on clinical evolution) and immunosuppressive agents such as methotrexate, azathioprine, mycophenolate mofetil, or biological agents such as rituximab [6]. Integration into a rehabilitation program can enhance the functional prognosis by improving muscle strength and performance [1,2], preventing disuse atrophy [1], improving aerobic and functional capacity, and decreasing disease activity [2]. The molecular mechanisms of these effects are not completely known but can be partially explained by the inhibition of genes associated with inflammation and fibrosis and the upregulation of genes associated with aerobic metabolism in muscle tissue [2].

## Case presentation

**Figure 1:**
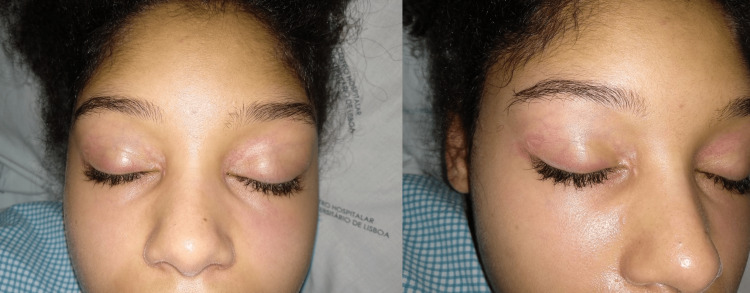
Heliotrope erythema. A pink, red, or purplish coloring around the eyes and eyelids, with or without edema [5].

**Figure 2:**
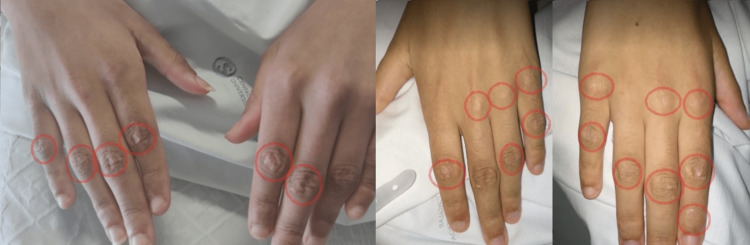
Grotton’s sign. Symmetrical, occasionally scaly, erythematous eruption over the extensor surfaces of the metacarpophalangeal and interphalangeal joints of the fingers. It may also involve the knees and elbows [5].

**Figure 3:**
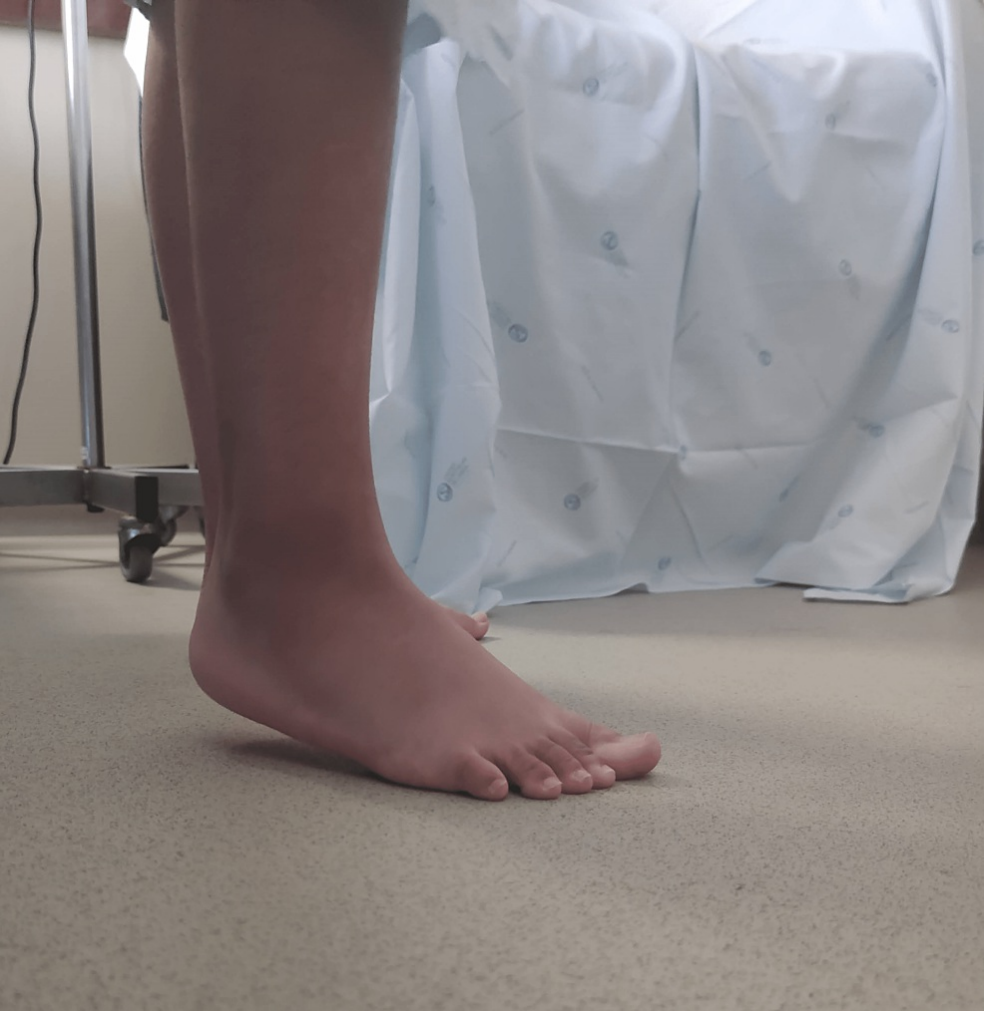
Ankle joint contracture. The patient is trying to place the heel on the ground but is unable to do it due to plantar flexion contracture.

**Figure 4:**
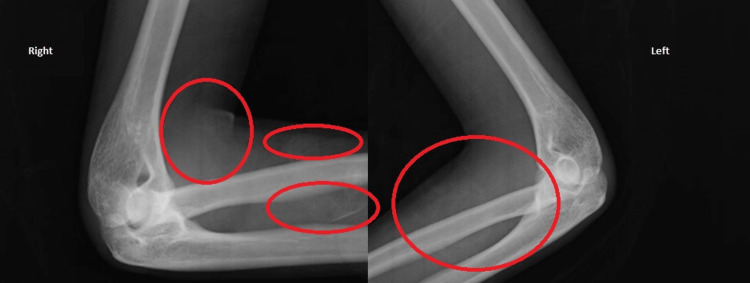
Elbow radiographs. Note the increased opacity of the peri-articular soft tissues, indicating the development of calcinosis.

A 19-year-old female presented with arthritis of the shoulders, elbows, wrists, and ankles. She had severe proximal asymmetrical muscle weakness, with compromised balance and gait, as well as respiratory muscle weakness, dysphonia, and dysphagia. Concomitantly, she presented heliotrope erythema on the eyelids (Figure 1) and Grotton’s sign on the interphalangeal joints of the hands (Figure 2). She had decreased range of motion in all of the affected joints (Figure 3). Functionally, she needed assistance in vertical transfers and gait. Radiographs documented the presence of calcinosis in the peri-articular soft tissue (Figure 4).

Laboratory tests showed elevated transaminases, gamma-glutamyl transferase, creatine kinase, myoglobin, and lactic dehydrogenase, with positive NXP-2 autoantibodies. Electromyography with nerve conduction studies revealed severe myopathy associated with signs of muscle fiber necrosis, and respiratory function tests documented moderate restrictive respiratory syndrome. Because the most likely diagnosis in the differential was DM, the patient was started on 500 mg of methylprednisolone/day for five days, with a later transition to 40 mg of prednisolone (1 mg/kg/day) and 50 mg of azathioprine/day, with improvement in muscle strength, dermatological manifestations, and swallowing symptoms. The diagnosis was later confirmed by muscle biopsy, with the presence of atrophy, necrosis, and regeneration of muscle fibers, as well as the presence of inflammatory cells in the perimysium, consistent with DM.

The patient was then transferred to a Physical Medicine and Rehabilitation inpatient department to a multidisciplinary rehabilitation program, encompassing physiotherapy, occupational therapy, and speech therapy. On physical examination, she presented dysphagia and dysphonia with generalized muscular atrophy, heliotrope erythema on the eyelids, and Grotton’s papules on the interphalangeal joints of the hands. She had limited passive and active range of motion, quantified by a goniometer. Muscle strength was assessed using the Manual Muscle Testing-8 (MMT-8) at admission, week three, and at discharge (week five). Functional Independence Measure (FIM) and Barthel Index (BI) were also documented (Table 1).

**Table 1:** FIM, BI, MMT-8 score, range of motion, and muscle strength evolution through the inpatient rehabilitation. The Functional Independence Measure (FIM) and Barthel Index (BI) scores were applied at admission, at three weeks, and on the patient’s discharge after five weeks of hospitalization. To assess the patient’s strength, the Manual Muscle Testing 8 (MMT-8) score was used. The range of motion was quantified by a goniometer.

	Admission		Third week		Fifth week	
FIM	86		102		118	
BI	50		75		90	
MMT-8 score	73		85		94	
	Right	Left	Right	Left	Right	Left
Range of motion						
Shoulder flexion	120°	100°	140°	130°	170°	170°
Shoulder abduction	90°	80°	150°	150°	180°	180°
Elbow extension	-20°	-30°	-15°	-20°	-10°	-15°
Forearm supination	90°	70°	90°	85°	90°	90°
Wrist flexion	60°	60°	75°	70°	80°	75°
Wrist extension	80°	80°	80°	85°	85°	85°
Hip flexion	100°	100°	115°	110°	120°	120°
Ankle dorsiflexion	-15°	-15°	0°	0°	5°	5°
Muscle strength						
Neck flexors	2		3		3	
Deltoid	2	2	4	4	4	4
Biceps brachii	6	6	6	8	7	8
Gluteus maximus	3	5	3	5	5	6
Gluteus medius	3	6	4	6	6	6
Quadriceps	6	6	7	8	8	8
Wrist extensors	6	6	6	6	7	6
Ankle dorsiflexors	7	7	7	8	8	8

During inpatient rehabilitation admission, the patient had weekly analytical reassessments and a progressive increase of azathioprine dose up to 100 mg/day. She also maintained follow-up with psychology and nutrition, optimizing her nutritional intake, with a favorable evolution. She was discharged with normal swallowing function and improved speech phonetics. She progressed to modified independence in transfers and gait, managing to go up and down the stairs with minimal help from a third person.

The patient was discharged with oral corticosteroid therapy, with a scheduled taper. Therapy was maintained with azathioprine 100 mg/day and treatment with calcium carbonate 1,500 mg and cholecalciferol 400 IU for prevention of secondary osteoporosis.

## Discussion

Recovery of muscle function does not occur in most patients with DM, leading to a negative impact on their quality of life. The present clinical case raised some questions regarding the efficacy, safety, and outcome of rehabilitation treatment.

Safety of rehabilitation

The institution of an early rehabilitation program appears to have beneficial effects, with a positive impact on functionality and independence. This fact contradicts the classic belief that performing exercise may enhance muscle inflammation and increase CK levels, aggravating muscle weakness and fatigue [1,4]. The increase in CK blood levels with consequent normalization after 24 hours is a normal acute response to exercise, but there are no studies that support this idea [4]. A review by Benlidayi et al. [7] found that exercise has a positive impact on idiopathic, autoimmune, and non-autoimmune myositis. These benefits include increased enzyme activity, mitochondrial biogenesis, reconditioning of inflammatory/immune pathways, decreased endoplasmic reticulum stress, modulation of gene expression, increased protein synthesis and cytoskeletal remodeling, and decreased muscle fibrosis and infiltration in nonmuscular tissues.

Thus, current evidence considers that it is safe to conduct a rehabilitation program [1,2,4] without negative effects on disease activity [2]. In some studies included in a systematic review, disease activity remained stable or even improved [2]. Thus, physiotherapy has a beneficial effect when there is symptomatic stability [2].

Some studies even suggest the institution of an early rehabilitation program in the presence of active disease, without increased risk of inflammation and exacerbation of the disease, in patients who went through a supervised exercise program [1,8]. Alexander et al. [9] evaluated the effects of a home exercise program in patients with PM and DM of recent onset, after four weeks of pharmacological therapy, noting that exercise in patients with active disease is safe. Therefore, a personalized exercise program early in the active disease may be recommended, with regular assessment of muscle performance and the overall health of patients.

Anti-inflammatory effect

The exercise demonstrated an anti-inflammatory effect [1,4] by increasing anti-inflammatory cytokines and reducing inflammatory cytokines. Some studies have demonstrated that exercise inhibits the expression of pro-inflammatory genes [10,11] and genes related to the fibrosis process [10], promoting the upregulation of anti-inflammatory and muscle growth genes [11].

Performance and aerobic function

According to some studies, exercise increases the performance of patients with DM by improving aerobic muscle metabolism [1,4,10-14] increasing maximum VO_2_ values, ​​and improving mitochondrial enzymatic activity and oxygen supply from the skeletal muscle. In one of these studies, Munter et al. [11] found that there was an activation of the aerobic phenotype and a decreased inflammatory response, through the positive regulation of molecular pathways involved in aerobic capacity, muscle remodeling, and increased capillary density, improved mitochondrial function, and ATP production.

Munters et al. [13], performed a clinical study of outpatient rehabilitation three times per week for 12 weeks. Each rehabilitation session included three components, namely, aerobic conditioning, muscle strengthening, and myotendinous stretching. It started with stationary cycling for 35 minutes, five minutes until reaching 50% of maximum VO_2_, and 30 minutes until reaching 70% of maximum VO_2_. Subsequently, it included a 20-minute period of resistance exercises, with 30-40% of the patient’s maximum repetition, followed by myotendinous stretching.

Alexanderson et al. [15] evaluated the benefits of an intensive outpatient rehabilitation program in patients with PM and DM, three times per week for seven weeks. The program included muscle strengthening with 10 repetitions of resistance exercises encompassing the deltoids, quadriceps, latissimus dorsi muscles, biceps brachii, gastrocnemius, and cervical flexors. They concluded that these patients can perform more intense resistance exercises, with benefits in their performance and without increasing muscle inflammation.

Muscle strength

The literature suggests that the institution of a rehabilitation program leads to an improvement in muscle strength [1,2,4,8,12,14]. In muscle strengthening and aerobic training, concentric contraction type is advised due to the possibility of muscle damage by eccentric contraction [1]. High-intensity muscular resistance training is not recommended in patients with DM due to microscopic damage to muscle fibers [1]. Some studies consider that an isolated muscle-strengthening program may have little or no effect, as it should be associated with aerobic exercise [12].

Combining low-intensity resistance training with blood flow restriction may induce similar gains in muscle mass and strength compared to high-intensity resistance training, without as much risk of muscle damage [1]. Minniti et al. [16] evaluated the efficacy and safety of a blood flow restriction program in a systematic review including patients with various musculoskeletal pathologies, namely, PM, DM, IBM, thoracic outlet syndrome, Achilles tendon rupture, and bone fractures. They concluded that this program seems to be a safe method of muscle strengthening in musculoskeletal pathologies of the knee, requiring further studies in other pathologies. In addition, a better definition of adverse effects is needed to differentiate between common, uncommon, and rare effects, and further studies are needed to screen patients who may be at an increased risk of adverse effects.

Functionality and quality of life

The previously mentioned effects translate into an improvement in the functionality of activities of daily living, capacity, and quality of life of the patients [8,10]. These outcomes were studied both in patients with PM and DM [10], showing that physical exercise demonstrated a reduction in disability and limitation of activities, as well as an improvement in quality of life and decreased disease activity [10,14]. The study even revealed promising results in open studies conducted in patients with IBM and juvenile DM, with a reduction of disability and limitation of activities and improvement in quality of life.

Elnaggar et al. [17] evaluated the effects of aquatic plyometric exercises in patients with juvenile DM in a blind randomized trial. The group that performed the exercises showed an improvement in muscle strength (hip flexors and abductors, knee flexors, and extensors), fatigue perception, functionality, and disease activity. The authors consider these exercises to be safe, feasible, well-tolerated, and useful in increasing muscle strength, reducing fatigue, and increasing functional capacity in this population. Sports activities such as cycling, walking, and jogging on flat ground are recommended [1].

Nutrition

Chung et al. [18] tested the hypothesis that oral supplementation with creatine could potentiate the effects of exercise in patients with DM or stabilized PM under chronic medical therapy. Supplementation was 20 g per day for eight days followed by 3 g per day for six months, and it was found that supplementation potentiates the effects of exercise and improves the ability to perform high-intensity exercise, with no adverse effects.

Exercise recommendations in DM

Despite the fact that in the last 15 years there has been intensive research with accumulated evidence supporting the benefits of exercise in patients with established or newly diagnosed DM, no exercise program appears to be superior. Alexanderson et al. [19] concluded that exercise recommendations should be similar to those of the healthy population (Table 3).

**Table 2:** Exercise recommendations for the general population. According to the American College of Sports Medicine, the general exercise recommendation program should include three components, namely, muscle strengthening, muscular endurance, and aerobic capacity [19].

Goal of exercise	Duration (minutes)	Frequency (days/week)	Intensity (% of one maximal repetition)	Intensity (% of maximal age, adjusted HR)
Muscle strength improvement	-	2	60–80%	-
Muscular endurance improvement	-	2	30–40%	-
Aerobic capacity improvement	30–60	3	-	60–85%

## Conclusions

DM is an insidious chronic disease with multisystemic involvement and a great loss of independence. Its treatment is based on a multidisciplinary approach. Physical exercise is beneficial and safe in patients with inflammatory myopathy, although there is not enough evidence to support specific guidelines in these patients. Based on the available evidence, the recommendations do not differ considerably for the healthy population, and the established rehabilitation program must be individualized and directed to the deficits and limitations of each patient. The present work emphasizes the need for systemic evaluation and individualization of the rehabilitation program, as well as the importance of immunosuppressed patients being at risk of infectious complications and pharmacological adverse effects of the therapy, namely, osteoporosis secondary to high-dose corticosteroid therapy or corticosteroid-induced myopathy. Clinical follow-up should be regular with monitoring during the hospitalization period, and the rehabilitation program should be adapted based on the subjective perception of exertion. Because most subgroups of myositis are rare, most studies include small samples, and in some publications, the duration, frequency, and intensity of exercise are not specifically reported. Additional larger studies are needed to deepen the knowledge of the mechanisms that cause disability, the effects of exercise, and which exercise program is more effective in patients with different entities in the group of idiopathic myositis.
